# Common mental disorders prevalence in adolescents: A systematic review and meta-analyses

**DOI:** 10.1371/journal.pone.0232007

**Published:** 2020-04-23

**Authors:** Sara Araújo Silva, Simoni Urbano Silva, Débora Barbosa Ronca, Vivian Siqueira Santos Gonçalves, Eliane Said Dutra, Kênia Mara Baiocchi Carvalho

**Affiliations:** 1 Graduate Program in Human Nutrition, University of Brasilia, Federal District, Brasilia, Brazil; 2 Graduate Program in Collective Health, University of Brasilia, Federal District, Brasilia, Brazil; University of the Witwatersrand, SOUTH AFRICA

## Abstract

An increasing number of original studies suggest the relevance of assessing mental health; however, there has been a lack of knowledge about the magnitude of Common Mental Disorders (CMD) in adolescents worldwide. This study aimed to estimate the prevalence of CMD in adolescents, from the General Health Questionnaire (GHQ-12). Only studies composed by adolescents (10 to 19 years old) that evaluated the CMD prevalence according to the GHQ-12 were considered. The studies were searched in Medline, Embase, Scopus, Web of Science, Lilacs, Adolec, Google Scholar, PsycINFO and Proquest. In addition, the reference lists of relevant reports were screened to identify potentially eligible articles. Studies were selected by independent reviewers, who also extracted data and assessed risk of bias. Meta-analyses were performed to summarize the prevalence of CMD and estimate heterogeneity across studies. A total of 43 studies were included. Among studies that adopted the cut-off point of 3, the prevalence of CMD was 31.0% (CI 95% 28.0–34.0; I^2^ = 97.5%) and was more prevalent among girls. In studies that used the cut-off point of 4, the prevalence of CMD was 25.0% (CI 95% 19.0–32.0; I^2^ = 99.8%). Global prevalence of CMD in adolescents was 25.0% and 31.0%, using the GHQ cut-off point of 4 and 3, respectively. These results point to the need to include mental health as an important component of health in adolescence and to the need to include CMD screening as a first step in the prevention and control of mental disorders.

## Introduction

Common Mental Disorders (CMD) refer to depressive and anxiety disorders and are distinct from the feeling of sadness, stress or fear that anyone can experience at some moment in life. Despite some methodological differences in the epidemiological studies, it is estimated that 4.4% and 3.6% of the world adult population suffers from depressive and anxiety disorders, respectively [1]. CMD can affect health and quality of life, and it is noted that CMD affect people at an early age [2].

The Global Burden of Diseases, Injuries, and Risk Factors (GBD) study is a comprehensive study that evaluates incidence, prevalence, and years lived with disability (YLDs), which in its most recent study evaluated the period from 1990 to 2017 for 195 countries and territories, and identified that the burden of mental disorders is present for males and females and across all age groups. The findings of the GDB indicate that mental disorders have consistently formed more than 14% of age-standardized YLDs for nearly three decades, and have greater than 10% prevalence in all 21 GBD regions [3]. Mental disorders are not often correctly identified and have negative consequences on people's health.

At the population level the use of self-report psychiatric screening instruments, such as the General Health Questionnaire (GHQ), has been recommended to track CMD, also known as psychological distress/problems or psychiatric morbidity or non-psychotic mental illnesses [4]. The GHQ-12 is a short and self-report form to identify people with psychological distress or CMD [5,6]. This validated instrument comprising a multidimensional evaluation based in three factors: anxiety and depression, social dysfunctions and loss of confidence [7] and can be applied in individuals of different ages [8].

Adolescence, defined as a transitional phase between ages 10 and 19 [9] is generally perceived as a phase of life with no health problems. However, approximately 20% of adolescents experience a mental health problem, most commonly depression or anxiety [10].

Although there are preliminary data on the severity of these conditions among adolescents [11], there has been a lack of knowledge about the magnitude of CMD in adolescents worldwide. There was a systematic review of the global prevalence of CMD, published in 2014, which incorporated studies from 1980 to 2013 that surveyed people aged 16 to 65 and using diagnostic criteria other than GHQ. In addition, from this study it was not possible to identify the prevalence of CMD in adolescents [12]. In this context, a systematic review of the literature was carried out to estimate the prevalence of CMD in adolescents around the world, from item 12 of the GHQ.

## Materials and methods

This systematic review followed the Preferred Reporting Items for Systematic Review and Meta-analyses PRISMA checklist [13] and for meta-analyses followed Meta-analysis of Observational Studies in Epidemiology (MOOSE) [14] guidelines.

### Protocol and registration

The systematic review protocol was registered in the International Prospective Register of Systematic Reviews (PROSPERO), registration number CRD42018094763.

### Eligibility criteria

The present study included observational studies. Only studies that assessed the prevalence of CMD according to GHQ-12 in adolescents (10 to 19 years old) were considered for retention. In studies that evaluated adolescents and also individuals outside the age group of interest for this review, an attempt was made to identify only those eligible through the information contained in the article or by contacting authors.

Moreover, no restrictions of language, publication date or status were applied. Studies of specific groups such as obese or diabetic individuals, adolescents in treatment of any health condition, college students, people who had traumatic experiences, pregnant teenagers and people with physical disabilities were not eligible. The ineligibility criterion considered those conditions that predispose to a higher risk of CMD, such as life events that presumably increase the chances of having feelings of stress, depression or anxiety. For example, among college students depression rates could be substantially higher than those found in the general population, probably because they experience moments of stress related to studies or future choices involving the profession phase of life [15]. Systematic reviews, interventional studies or ecological estimates were also not included.

### Information sources

A systematic search of the following databases was conducted to identify relevant studies: Medline, Embase, Scopus, Web of Science, Lilacs and Adolec. A partial grey literature search was also performed in Google Scholar, PsycINFO and Proquest Dissertation and Theses. The Google Scholar search was limited to the first 200 most relevant articles. The search was conducted on December 1, 2018 and updated in April 1, 2019. Additional articles, were hand-searched in selected articles to identify potentially eligible studies not retrieved by the database search. The search strategy was reviewed by two researchers, one of them with extensive experience in systematic reviews, according to the criteria of the checklist of the Peer Review of Electronic Search Strategies (PRESS checklist) [16].

The following strategy was adapted for the databases: (Adolescent OR Teenager OR Child OR Young OR Teen OR Youth OR Juvenile OR Adolescence OR Younger) AND (“General Health Questionnaire” OR GHQ OR GHQ-12) AND (“common mental disorders” OR CMD OR Anxiety OR anxious OR depression OR dysthymia OR “generalized anxiety disorder” OR “panic disorder” OR phobia OR “social anxiety disorder” OR “obsessive-compulsive disorder” OR “mental disorder” OR “mental health” OR "Psychological stress" OR "Life Stress" OR "Psychologic Stress" OR "Mental suffering" OR Anguish OR "Emotional stress") AND (Survey OR “Cross-sectional studies” OR Prevalence OR frequency OR "Cross-sectional" OR Observational). More information on the search strategies is provided in S1 Appendix. The Covidence Software (Cochrane Collaboration software®, Melbourne, Australia) was used to remove duplicate references and for the screening procedure, applied independently.

### Data collection process

The study selection process was carried out in two stages. First, the articles were selected based on their titles and abstracts, followed by a full text assessment. These two stages were carried by two independent authors (SAS and SUS) and the records that did not meet the inclusion criteria were discarded. The disagreements were resolved by consensus and counted on the participation of a third author (DBR).

Data were extracted in duplicate by authors and discrepancies were resolved by consensus. The following data were collected: authors, year of publication, year of research, country, study design, age (mean or range), sample size (sex), GHQ cut-off point and outcome of the studies (prevalence of CMD). The corresponding authors of the studies were contacted (at least two attempts of contact) in case of unavailable data.

The 12-item version of the GHQ has psychometric properties comparable to those of the longer versions of the questionnaire and the items of this instrument describe positive and negative aspects of mental health in the last two weeks and present a scale with four response options. The difference in the scale for positive and negative items indicates that the higher the score, the higher level of psychiatric disorders. The studies show great variation in the scoring methods for the GHQ, with scales ranging from zero to 12 or zero to 36.

### Risk of bias within individual studies

The critical appraisal tool, recommended by The Joanna Briggs Institute for cross-sectional studies, was used to assess the risk of bias. The purpose of this appraisal is to assess the methodological quality of a study and to determine the possibility of bias in its design, conduct and analysis. This instrument consists of nine questions answered as “yes”, “no”, “unclear”, or “not applicable” [17].

For this study, when all items were answered “yes”, the risk of bias were considered low, and if any item were classified as “no” or “unclear”, a high risk of bias were expected. No scores were assigned; results were expressed by the frequency of each classification of the evaluation parameters. These ratings were not used as a criterion for study eligibility.

### Summary measures and data analysis

The primary outcome was the prevalence of CMD, with a confidence interval of 95% (CI 95%). We estimated the summary measures for the total population and subgroups defined by sex, risk of bias and income level according to the World Bank classification [18]. The meta-analyses were calculated using a random-effect model and weighed by the inverse of the variance. The heterogeneity was evaluated by the Chi-square test with significance of p<0.10, and its magnitude was determined by the I-squared (I^2^) [19].

Meta-regressions were performed in order to identify possible causes of heterogeneity using the Knapp and Hartung test [20] with the following variables: risk of bias, sample size, proportion of female adolescent, year of study and income level. The small-study effect by visual inspection of the funnel graph and Egger's test [21] was also evaluated.

Analyzes were performed with the "Metaprop" command of the Stata software (version 14.0), adopting p<0.05.

## Results

### Study selection

**Fig 1:**
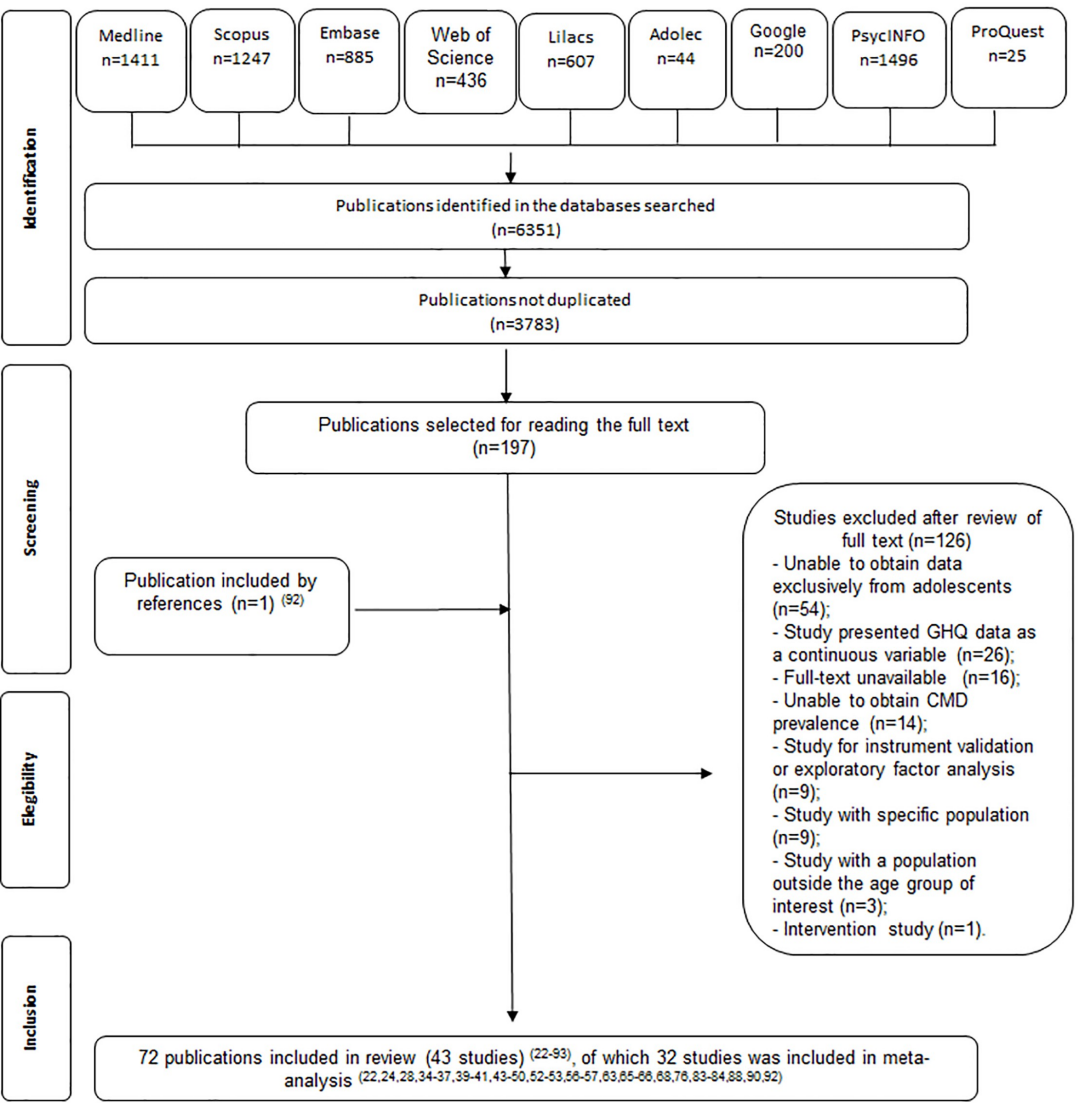
Flow chart of systematic review procedure for illustrating search results, selection and inclusion of studies. *Adapted from PRISMA.

A total of 6 351 articles were initially found in the nine electronic databases, including grey literature. After removing the duplicates, the titles and abstracts of 3 783 articles were screened, and 197 potentially relevant studies were selected for full-text reading. An additional record was selected from the reference lists of the fully read articles. A total of 126 articles were excluded for nominated reasons (see S1 Table). Forty-three studies (reported in 72 articles) [22–93] were therefore selected for inclusion in this review. The screening process is detailed in Fig 1.

### Study characteristics

Table 1 shows a summary of the study characteristics. A total of 43 studies (200 980 participants; 19 countries) were included. The CMD prevalence studies were conducted in Asia [26,27,34,39,40,45,48–50,52–54,57,70,89,90], America [38,41,44,84], Africa [22], Europe [24,28,32,35–37,43,46,47,56,63,65,68,71,76,88,92] and Oceania [66,83]. The majority of studies (n = 33) had a cross-sectional design.

**Table 1:** Summary of characteristics of included studies.

Author, year	Year of research	Country	Study design	Age (mean or range)	Sample size (sex)	GHQ^α^ cut-off point
Amoran, 2005^1^	NI	Nigeria	Cross-sectional	15 to 19	197	3^b^
Arun, 2009	NI	India	Cross-sectional	12 to 19	2 402 (boys = 1 371; girls = 1 031)	3^b^
Augustine, 2014	2009–2010	India	Cross-sectional	15 to 19	145 (all boys)	3^b^
Ballbè, 2015^2^	2011–2012	Spain	Cross-sectional	15 to 19	740 (boys = 396; girls = 344)	3^b^
Bansal, 2009	NI	NI	Cross-sectional	NI (9th grade students)	125	14^c^
Cheung, 2011	NI	China	Cross-sectional	14.70±2.02	719 (boys = 434; girls = 285)	11^c^
Czaba£a, 2005^3^	2002	Poland	Cross-sectional	13.8	1 123 (boys = 521; girls = 600)	3^b^
Dzhambov, 2017^4^	2016	Bulgaria	Cross-sectional	15 to 19	557 (boys = 408; girls = 149)	3^b^
Emami, 2007	2004	Iran	Cross-sectional	17 to 18	4 310 (boys = 1 923; girls = 2 387)	7^b^
Fernandes, 2013	2006	India	Cross-sectional	16 to 18	1 488	5^b^
Gale, 2004^5^	1986	United Kingdom	Longitudinal	16 (range not available)	5 187 (boys = 2 222; girls = 2 965)	3^b^
Gecková, 2003^6^	1998	Slovakia	Cross-sectional	15 (range not available)	2 616 (boys = 1 369; girls = 1 243)	2/3^b^^,^^c^
Glendinning, 2007	2002–2003	Russia	Cross-sectional	14 to 15	626	4^b^
Gray, 2008	1998 and 2003	United Kingdom	Cross-sectional	13 to 15	1 253	4^b^
Green, 2018	2017–2013	United Kingdom	Longitudinal	16 (range not available)	1 204 (boys = 619; girls = 585)	3^b^
Hamilton, 2009	2005	Canada	Cross-sectional	12 to 19	4 078 (boys = 2 092; girls = 1 986)	6^b^
Hori, 2016	2011	Japan	Cross-sectional	12 to 19	744 (boys = 373; girls = 371)	4^b^
Kaneita, 2009	2004	Japan	Longitudinal	13 to 15	516 (boys = 294; girls = 222)	4^b^
Lopes, 2016^7^	2013–2014	Brazil	Cross-sectional	12 to 17	74 589 (boys = 33 364; girls = 41 225)	3^b^
Mäkelä, 2015	2008	Finland	Cross-sectional	15 to 19	225 (boys = 102; girls = 123)	4^b^
Mann, 2011	2007	Canada	Cross-sectional	12 to 19	3 311 (boys = 1 566; girls = 1 745)	3^b^
McNamee, 2008	2005	Ireland	Cross-sectional	16 (range not available)	868 (boys = 352; girls = 516)	4^b^
Miller, 2018	2018	United Kingdom	Longitudinal	13 to 17	407 (boys = 204; girls = 203)	4^b^
Munezawa, 2009	NI	Japan	Cross-sectional	12 to 14	916 (boys = 568; girls = 348)	4^b^
Nakazawa, 2011	2008	Japan	Cross-sectional	12 to 15	4 864 (boys = 2,429; girls = 2,435)	4^b^
Nishida, 2008^8^	2006	Japan	Cross-sectional	12 to 15	4 894 (boys = 2 523; girls = 2 371)	4^b^
Nur, 2012	2009–2010	Turkey	Cross-sectional	15 to 19	244 (all girls)	4^b^
Ojio, 2016	2006	Japan	Cross-sectional	12 to 18	15 637 (boys = 7 953; girls = 7 684)	4^b^
Oshima, 2010^9^	2009	Japan	Cross-sectional	12 to 18	341 (boys = 173; girls = 168)	5^b^
Oshima, 2012^10^	2008–2009	Japan	Cross-sectional	12 to 18	17 920 (boys = 8 886; girls = 9 034)	4^b^
Padrón, 2012^11^	2008–2009	Spain	Cross-sectional	15 to 17	4 054 (boys = 1 951; girls = 2 103)	3^b^
Pisarska, 2011	2004	Poland	Cross-sectional	15 to 16	722 (boys = 383; girls = 335)	3^b^
Rickwood, 1996	1994	Australia	Longitudinal	16 to 19	4 163 (boys = 1 988; girls = 2 175)	4^b^
Rothon, 2012^12^	2005	United Kingdom	Longitudinal	14 to 15	13 539 (boys = 7 852; girls = 7 579)	4^b^
Roy, 2014	2009–2010	India	Cross-sectional	14 to 15 (around 80% of sample)	400 (boys = 200; girls = 200)	15^c^
Sweeting, 2009^13^	1987	United Kingdom	Longitudinal	15.8±3.5 months	505	2/3; 3/4;4/5^b^
Sweeting, 2009^13^	1999	United Kingdom	Longitudinal	15.5±3.6 months	2 196	2/3; 3/4;4/5^b^
Sweeting, 2009^13^	2006	United Kingdom	Longitudinal	15.5±3.8 months	3 194	2/3; 3/4;4/5^b^
Thomson, 2018^14^	1991–2014	United Kingdom	Cross-sectional	16 to 19	11 397 (boys = 5 376; girls = 6 021)	4^b^
Trainor, 2010	2001	Australia	Longitudinal	13 to 17	947 (boys = 390; girls = 557)	4^b^
Trinh, 2015^15^	2009	Canada	Cross-sectional	15,8	2 660 (boys = 1 236; girls = 1 397)	3^b^
Van Droogenbroeck, 2018	2008	Belgium	Cross sectional	15 to 19	680 (boys = 341; girls = 339)	4^b^
Yusoff, 2010	NI	Malaysia	Cross-sectional	16 (range not available)	90 (boys = 40; girls = 50)	4^b^

NI: Not informed.

^α^GHQ: General Health Questionnaire, 12 items.

^b^The score range was 0–12.

^c^The score range was 0–36.

^1^Amoran, 2007

^2^(Basterra, 2017; Gotsens, 2015)

^3^Bobrowski, 2007

^4^Dzhambov, 2018

^5^(Steptoe, 1996; Collishaw, 2010; Morgan, 2012)

^6^Gecková, 2004

^7^Telo, 2018

^8^Nishida, 2010

^9^Yamasaki, 2018

^10^(Kinoshita, 2011; Ando, 2013; Shiraishi, 2014; Kitawaga, 2017; Morokuma, 2017)

^11^Padrón, 2014

^12^Hale, 2014

^13^(West, 2003; Young, 2004; Sweeting, 2008; Sweeting 2010)

^14^(Fagg, 2008; Lang, 2011; Maheswaran, 2015; Pitchfort, 2016 and 2018)

^15^(Hamilton, 2011; Arbour-Nicitopoulos, 2012; Isaranuwatchai, 2014).

For the purpose of comparing the studies, we selected only those that presented the score scale from zero to 12, totaling 32 studies classified by 3 or 4 diagnostic cut-off points. Thus for the set of studies that adopted the cut-off point of 3 or more symptoms of the GHQ-12, the sample size varied from 145 adolescents in India [45] to 74 589 in Brazil [41], these studies included 96 842 adolescents between the ages of 12 and 19 years. In the set of studies with cut-off point of 4 or more symptoms, it ranged from 90 adolescents in Malaysia [90] to 17 920 in Japan [57] and the total sample was 79 892 adolescents aged 12 to 19 years.

### Results of individual studies and synthesis of results

Only six (18.8%) studies were considered to be of low risk of bias. Considering that the GHQ is a self-administered instrument composed of validated questions and translated in several languages, the parameter that deals with the identification of the outcomes measured in a valid way was met by all the studies.

**Fig 2:**
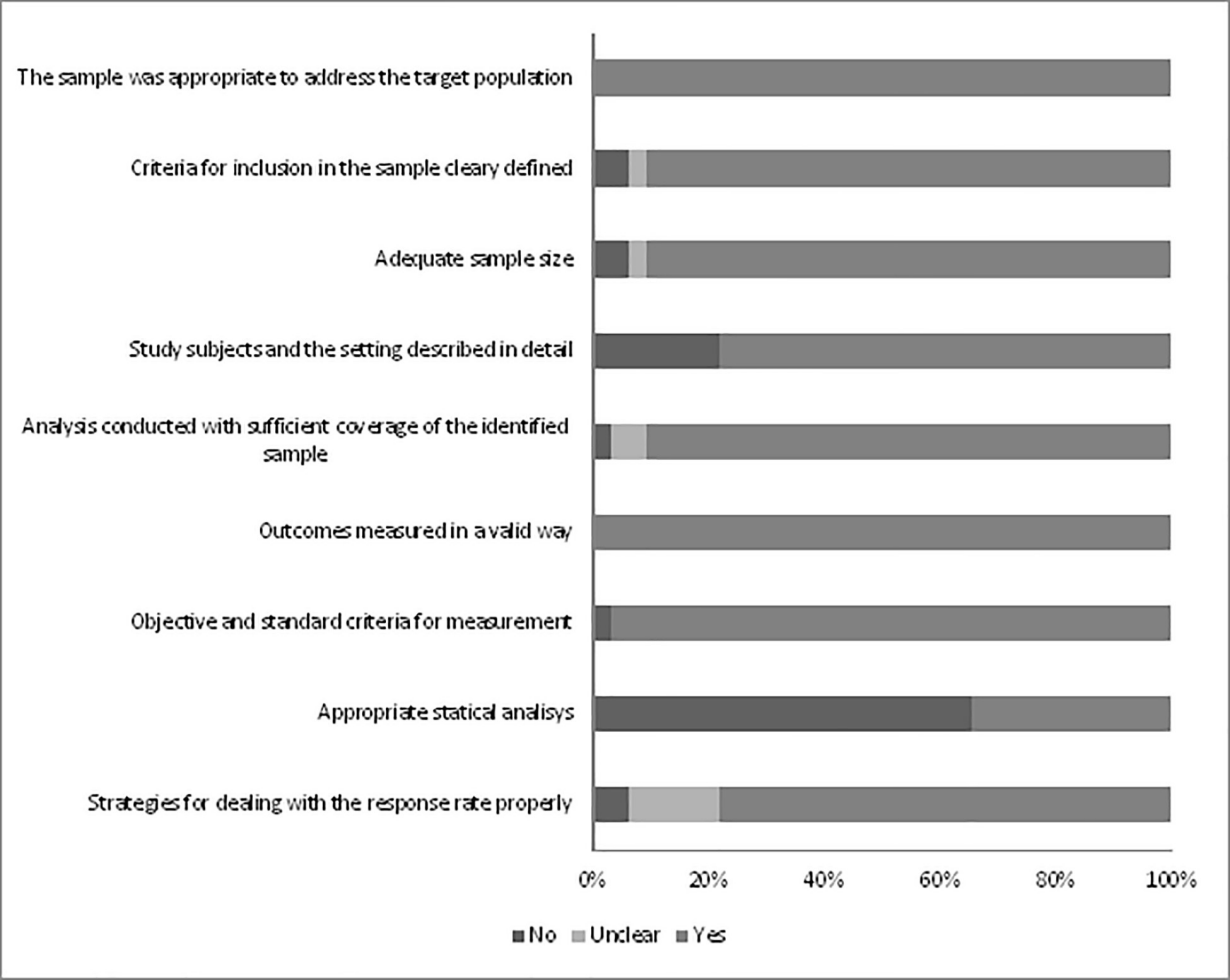
Risk of bias in the included studies (The Joanna Briggs Institute Critical Appraisal checklist for prevalence studies).

Two parameters were not met by most studies: (1) appropriate statistical analysis; and (2) study subjects and the setting described in detail (Fig 2 and Table 2). It is important to emphasize that the critical appraisal tool recommends that the numerator and the denominator be clearly reported, and that the percentages should be given with confidence intervals, so in the methods section there must be enough details to identify the analytical technique used and how specific variables were measured in the study. In addition, the study sample should be described in enough detail so that other researchers can determine if it is comparable to the population of interest to them. It is worth mentioning that some studies have reported the year of data collection and characteristics of the study population.

**Table 2:** Risk of bias for each individual study assessed by Joanna Briggs Institute critical appraisal checklist for prevalence studies.

_Studies_	_Criteria_								
	_1*_	_2*_	_3*_	_4*_	_5*_	_6*_	_7*_	_8*_	_9*_
_Amoran, 2005_	_Y_	_Y_	_N_	_Y_	_U_	_Y_	_Y_	_N_	_Y_
_Arun, 2009_	_Y_	_Y_	_Y_	_Y_	_Y_	_Y_	_Y_	_N_	_Y_
_Augustine, 2014_	_Y_	_Y_	_Y_	_N_	_Y_	_Y_	_Y_	_N_	_U_
_Ballbè, 2015_	_Y_	_Y_	_Y_	_Y_	_Y_	_Y_	_Y_	_N_	_Y_
_Czaba£a, 2005_	_Y_	_Y_	_Y_	_Y_	_Y_	_Y_	_Y_	_N_	_Y_
_Droogenbroeck, 2018_	_Y_	_Y_	_Y_	_Y_	_Y_	_Y_	_Y_	_Y_	_N_
_Dzhambov, 2017_	_Y_	_Y_	_Y_	_Y_	_N_	_Y_	_Y_	_N_	_Y_
_Fagg, 2008_	_Y_	_Y_	_Y_	_Y_	_Y_	_Y_	_Y_	_N_	_Y_
_Gale, 2004_	_Y_	_Y_	_Y_	_Y_	_Y_	_Y_	_Y_	_N_	_Y_
_Glendinning, 2007_	_Y_	_Y_	_Y_	_Y_	_Y_	_Y_	_Y_	_N_	_Y_
_Green, 2018_	_Y_	_Y_	_Y_	_Y_	_Y_	_Y_	_Y_	_Y_	_U_
_Hori, 2016_	_Y_	_Y_	_Y_	_Y_	_Y_	_Y_	_Y_	_N_	_Y_
_Kaneita, 2009_	_Y_	_Y_	_Y_	_Y_	_Y_	_Y_	_Y_	_N_	_Y_
_Lopes, 2016_	_Y_	_Y_	_Y_	_Y_	_Y_	_Y_	_Y_	_Y_	_Y_
_Mäkelä, 2014_	_Y_	_U_	_Y_	_N_	_Y_	_Y_	_Y_	_N_	_Y_
_Mann, 2011_	_Y_	_Y_	_Y_	_Y_	_Y_	_Y_	_Y_	_Y_	_Y_
_McNamee, 2008_	_Y_	_Y_	_Y_	_N_	_Y_	_Y_	_Y_	_N_	_N_
_Miller, 2018_	_Y_	_Y_	_Y_	_N_	_Y_	_Y_	_Y_	_Y_	_U_
_Munezawa, 2009_	_Y_	_Y_	_Y_	_N_	_Y_	_Y_	_Y_	_Y_	_Y_
_Nakazawa, 2011_	_Y_	_Y_	_Y_	_N_	_Y_	_Y_	_Y_	_N_	_Y_
_Nishida, 2008_	_Y_	_Y_	_Y_	_Y_	_Y_	_Y_	_Y_	_N_	_Y_
_Nur, 2012_	_Y_	_Y_	_Y_	_Y_	_Y_	_Y_	_Y_	_Y_	_Y_
_Ojio, 2016_	_Y_	_Y_	_Y_	_Y_	_Y_	_Y_	_Y_	_N_	_Y_
_Oshima, 2012_	_Y_	_N_	_N_	_Y_	_Y_	_Y_	_Y_	_Y_	_Y_
_Padrón, 2012_	_Y_	_Y_	_Y_	_Y_	_Y_	_Y_	_Y_	_Y_	_Y_
_Pisarska, 2011_	_Y_	_Y_	_Y_	_Y_	_Y_	_Y_	_Y_	_Y_	_Y_
_Rothon, 2012_	_Y_	_Y_	_Y_	_Y_	_Y_	_Y_	_Y_	_N_	_Y_
_Thomson, 2018_	_Y_	_Y_	_Y_	_Y_	_U_	_Y_	_Y_	_N_	_U_
_Trainor, 2010_	_Y_	_Y_	_Y_	_Y_	_Y_	_Y_	_Y_	_N_	_U_
_Trinh, 2015_	_Y_	_Y_	_Y_	_Y_	_Y_	_Y_	_Y_	_Y_	_Y_
_Yusoff, 2010_	_Y_	_N_	_U_	_N_	_Y_	_Y_	_N_	_N_	_Y_
_Rickwood, 1996_	_Y_	_Y_	_Y_	_Y_	_Y_	_Y_	_Y_	_N_	_Y_

*Y = Yes, N = No, U = Unclear, NA = Not applicable

^1*^The sample was appropriate to address the target population

^2*^Criteria for inclusion in the sample cleary defined

^3*^Adequate sample size

^4*^Study subjects and the setting described in detail

^5*^Analysis conducted with sufficient coverage of the identified sample

^6*^Outcomes measured in a valid way

^7*^Objective and standard criteria for measurement

^8*^Appropriate statistical analysis

^9*^Strategies for dealing with the response rate properly

### Results of individual studies

**Fig 3:**
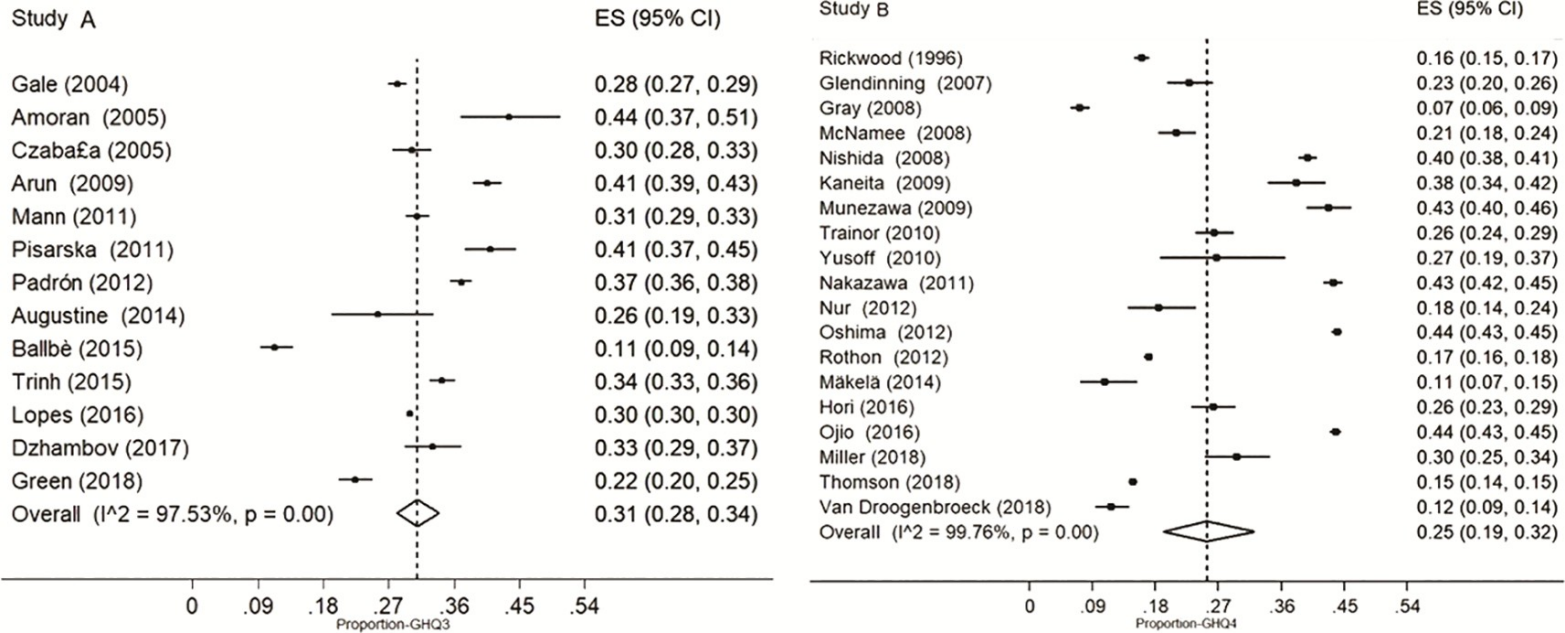
Common mental disorders prevalence in adolescents in studies with cut-off point 3 or more symptoms (A) and cut-off point 4 or more symptoms (B).

Among those that adopted the cut-off point of 3 or more symptoms, the prevalence of CMD was 31.0% (CI95% 28.0–34.0; I^2^ = 97.5%). In studies that used the cut-off point of 4 or more symptoms, the prevalence of CMD was 25.0% (CI 95% 19.0–32.0; I^2^ = 99.8%) (Fig 3). In the subgroup analysis, the heterogeneity remained high and it was observed that CMD is higher in female adolescents when considered the cut-off point 3 (Table 3).

**Table 3:** Prevalence of common mental disorders, by subgroups, in adolescents.

Subgroups	Number of studies	Number of participants	Prevalence (%)	Confidence interval 95%	I^2^(%)
**Cut-off 3 or more symptoms**					
**Sex**					
Male	10	42 192	23.0	21.0–26.0	92.9*
Female	9	50 863	38.0	34.0–42.0	96.9*
**Risk of bias**					
High	8	11 506	32.0	29.0–35.0	97.3*
Low	5	85 336	30.0	17.0–45.0	98.2*
**Income Level**					
High income	8	19 247	29.0	24.0–34.0	98.0*
Low income	5	79 745	35.0	28.0–41.0	96.9*
**Cut-off 4 or more symptoms**					
**Sex**					
Male	9	26 006	14.0	7.0–22.0	99.6*
Female	9	26 881	27.0	15.0–40.0	99.8*
**Risk of bias**					
High	18	79 648	26.0	19.0–33.0	99.8*
Low	1	244	18.0	14.0–24.0	-
**Income Level**					
High income	16	78 932	26.0	19.0–33.0	99.8*
Low income	3	960	22.0	18.0–26.0	-

*p < 0.001.

In the meta-regression, the high heterogeneity could not be explained by the studied variables: sex, income level and year of publication (p>0.05; data not shown).

**Fig 4:**
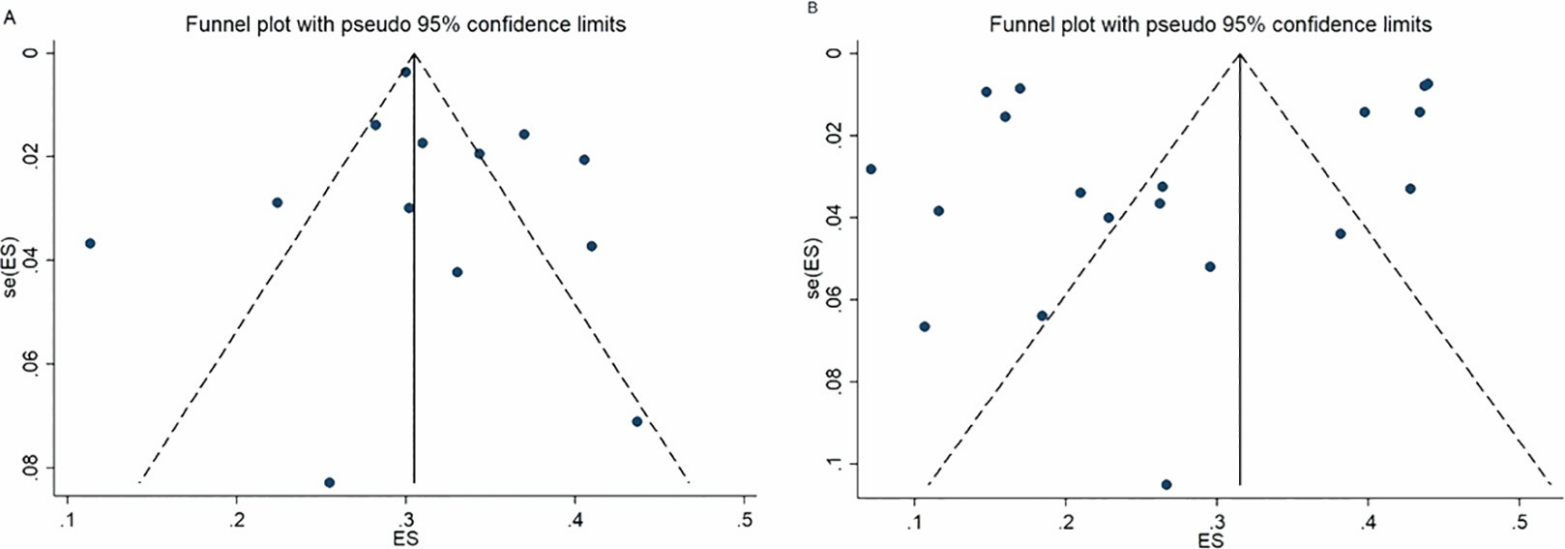
Funnel graph on the prevalence of common mental disorders in adolescents in studies with cut-off point 3 or more symptoms (A) and cut-off point 4 or more symptoms (B). Egger´s test: p<0.001.

The funnel graph was able to show the asymmetry between the studies, with greater representation of large studies (Fig 4). Graph A shows the studies that adopted cut-off point 3 and graph B, those that used cut-off point 4. Both illustrate that there is an effect of small studies and these findings were confirmed by the Egger's Test (p<0.001).

## Discussion

This systematic review was able to reveal the magnitude of CMD in adolescents from all over the world. When presented at this stage of life, CMD can have negative consequences throughout the future years. The problem is common and worrying, so much has been widely studied since the 1980s [12] however, they refer to studies with diverse populations and with different ways of identification of CMD.

Mental health can be influenced by several factors. Socioeconomic characteristics [38,94–97]; characteristics of lifestyle [43,56,64,83,98–100] [43]and also characteristics related to affective relationships [101–103], have been the focuses of studies already performed in adolescents.

Our meta-analysis revealed that very large studies were conducted in Japan and United Kingdom. It was reported that children and adolescents in Japan have greater depressive tendencies and this condition may be growing each year in several countries [104]. In the United Kingdom, the assessment and monitoring of psychological distress among adolescents is a common practice and generally performed in longitudinal studies for more than two decades [105].The evidence indicates that the relationship between culture or personal values and mental disorders differs across cultures and age groups [106]. An approach that takes into account the differences in social and cultural contexts is necessary to understand the occurrence and phenomenology of CMD in epidemiological studies, since there is a relationship between them but that needs to be better clarifies in future studies.

Although with some degree of methodological issue in most studies, since less than 20% of the studies presented low risk of bias, the results of this study indicate that CMD affect girls more, considering only the studies that adopted cut-off point 3. Permanent concern with physical appearance, body dissatisfaction, exposure to sexualization may be one of the reasons that affect girls' mental health [107].

Another factor that apparently influences the presence of CMD is income level. Even though the results presented in this systematic review showed no difference between income level of the countries and CMD, further studies with this focus are needed in order to deepen the knowledge about the subject. Longitudinal studies such as the British Household Panel Survey (BHPS) and Longitudinal Study of Young People in England (LYSPE) demonstrate the impact of economic recession and poverty in populations by strong associations between socioeconomic variables and health outcomes [76,108–111].

Although the GHQ is a validated instrument for detecting CMD, the scoring scale and cut-off point are not consensual, which impairs comparison among studies. Meta-analyses in the present study were based on cut-off points 3 and 4, since they were more frequent among the studies.

In relation to age, studies are commonly defined to be representative of the population aged 15 years or more, however, it is also important to investigate the phenomenon of CMD among the younger population (10 to 14 years), since global epidemiological data consistently report that up to 20% of children and adolescents suffer from a disabling mental illness [112]. Particular attention should be paid to the most vulnerable adolescent population in order to create strategies based on scientific evidence [113]. This systematic review revealed the severity of the problem by the worldwide high prevalence of CMD among adolescents, using a standardized criterion of measurement, the GHQ-12.

### Study limitations

In this review some of the eligible studies showed association data and did not present the prevalence and the respective confidence intervals, nor did they present the description of the evaluated population. It is possible that this review did not include all relevant publications, either because the articles did not present sufficient information or because the authors were not located or, finally, because of unanswered communication attempts.

It is observed that the different cut-off points for the GHQ-12 adopted in the original studies were a complicating factor in the identification of cases of CMD and in the comparison among studies. Even if measures were taken to combine studies that were as comparable as possible, this review included studies conducted at different times and places and with varying methodologies. These characteristics are revealed in the heterogeneity between the studies, typically found in cross-sectional studies and, therefore, we performed a subgroup analysis and a meta-regression, but without success.

### Strengths of the study

In the elaboration of this systematic review, some steps were considered as the registration of protocol in PROSPERO, the use of the PRESS checklist, blind selection of studies, the adoption of updated analytical methods and a search strategy that enabled the capture of a large numbers of studies. An extensive search for studies was carried out in the literature sources, the grey literature, and the reference lists of the eligible articles. When necessary, the authors of potentially eligible studies were contacted to obtain extra data to carry out the meta-analyses. Moreover, this systematic review followed the PRISMA tool guide and the Meta-analysis of Observational Studies in Epidemiology (MOOSE) [14].

## Conclusion

The global prevalence of CMD in adolescents was 25.0% and 31.0%, using the GHQ cut-off point of 4 and 3, respectively. CMD was more prevalent among girls when observing studies that adopted a 3 cut-off point. These results point to the need to include mental health as an important component of health in adolescence and to the need to include CMD screening as a first step in the prevention and control of mental disorders.

## Supporting information

S1 Appendix PRISMA checklist. (DOC)

S2 Appendix Search strategy and databases. (DOC)

S1 Table Details of excluded studies. (DOC)

S1 Data (XLSX)
