# Supplementary material for: Common mental disorders prevalence in adolescents: A systematic review and meta-analyses
Source: PLoS One. 2020 Apr 23;15(4):e0232007. doi: 10.1371/journal.pone.0232007 (PMC7179924; doi:10.1371/journal.pone.0232007)
Supplement: S2 Appendix — (DOC) [file pone.0232007.s002.doc]

# S2 Appendix. Search strategy and databases.

| **Databases** | **Search strategy** |
| --- | --- |
| **MEDLINE** | ((("adolescent"[MeSH Terms] OR "adolescent"[All Fields]) OR ("adolescent"[MeSH Terms] OR "adolescent"[All Fields] OR "teenager"[All Fields]) OR ("child"[MeSH Terms] OR "child"[All Fields]) OR Young[All Fields] OR ("adolescent"[MeSH Terms] OR "adolescent"[All Fields] OR "teen"[All Fields]) OR ("adolescent"[MeSH Terms] OR "adolescent"[All Fields] OR "youth"[All Fields]) OR Juvenile[All Fields] OR ("adolescent"[MeSH Terms] OR "adolescent"[All Fields] OR "adolescence"[All Fields]) OR Younger[All Fields]) AND ("General Health Questionnaire"[All Fields] OR GHQ[All Fields] OR GHQ-12[All Fields])) AND ("common mental disorders"[All Fields] OR ("Cmd"[Journal] OR "cmd"[All Fields]) OR ("anxiety"[MeSH Terms] OR "anxiety"[All Fields]) OR ("anxiety"[MeSH Terms] OR "anxiety"[All Fields] OR "anxious"[All Fields]) OR ("depressive disorder"[MeSH Terms] OR ("depressive"[All Fields] AND "disorder"[All Fields]) OR "depressive disorder"[All Fields] OR "depression"[All Fields] OR "depression"[MeSH Terms]) OR ("dysthymic disorder"[MeSH Terms] OR ("dysthymic"[All Fields] AND "disorder"[All Fields]) OR "dysthymic disorder"[All Fields] OR "dysthymia"[All Fields]) OR "generalized anxiety disorder"[All Fields] OR "panic disorder"[All Fields] OR ("phobic disorders"[MeSH Terms] OR ("phobic"[All Fields] AND "disorders"[All Fields]) OR "phobic disorders"[All Fields] OR "phobia"[All Fields]) OR "social anxiety disorder"[All Fields] OR "obsessive-compulsive disorder"[All Fields] OR "mental disorder"[All Fields] OR "mental health"[All Fields] OR "Psychological stress"[All Fields] OR "Life Stress"[All Fields] OR "Psychologic Stress"[All Fields] OR "Mental suffering"[All Fields] OR ("stress, psychological"[MeSH Terms] OR ("stress"[All Fields] AND "psychological"[All Fields]) OR "psychological stress"[All Fields] OR "anguish"[All Fields]) OR "Emotional stress"[All Fields]) AND (("surveys and questionnaires"[MeSH Terms] OR ("surveys"[All Fields] AND "questionnaires"[All Fields]) OR "surveys and questionnaires"[All Fields] OR "survey"[All Fields]) OR "Cross-sectional studies"[All Fields] OR ("epidemiology"[Subheading] OR "epidemiology"[All Fields] OR "prevalence"[All Fields] OR "prevalence"[MeSH Terms]) OR ("epidemiology"[Subheading] OR "epidemiology"[All Fields] OR "frequency"[All Fields] OR "epidemiology"[MeSH Terms] OR "frequency"[All Fields]) OR "Cross-sectional"[All Fields] OR Observational[All Fields]) |
| **EMBASE** | (adolescent OR teenager OR child OR young OR teen OR youth OR juvenile OR adolescence OR younger) AND ('general health questionnaire' OR ghq OR 'ghq 12') AND ('common mental disorders' OR cmd OR anxiety OR anxious OR depression OR dysthymia OR 'generalized anxiety disorder' OR 'panic disorder' OR phobia OR 'social anxiety disorder' OR 'obsessive-compulsive disorder' OR 'mental disorder' OR 'mental health' OR 'psychological stress' OR 'life stress' OR 'psychologic stress' OR 'mental suffering' OR anguish OR 'emotional stress') AND (survey OR 'cross-sectional studies' OR prevalence OR frequency OR 'cross-sectional' OR observational) |
| **SCOPUS** | ( TITLE-ABS-KEY ( adolescent  OR  teenager  OR  child  OR  young  OR  teen  OR  youth  OR  juvenile  OR  adolescence  OR  younger )  AND  TITLE-ABS-KEY ( "General Health Questionnaire"  OR  ghq  OR  ghq-12 )  AND  TITLE-ABS-KEY ( "common mental disorders"  OR  cmd  OR  anxiety  OR  anxious  OR  depression  OR  dysthymia  OR  "generalized anxiety disorder"  OR  "panic disorder"  OR  phobia  OR  "social anxiety disorder"  OR  "obsessive-compulsive disorder"  OR  "mental disorder"  OR  "mental health" )  OR  TITLE-ABS-KEY ( "Psychological stress"  OR  "Life Stress"  OR  "Psychologic Stress"  OR  "Mental suffering"  OR  anguish  OR  "Emotional stress" )  AND  TITLE-ABS-KEY ( survey  OR  "Cross-sectional studies"  OR  prevalence  OR  frequency  OR  "Cross-sectional"  OR  observational ) ) |
| **WEB OF SCIENCE** | TOPIC:(Adolescent OR Teenager OR Child OR Young OR Teen OR Youth OR Juvenile OR Adolescence OR Younger) AND TOPIC: (“General Health Questionnaire” OR GHQ OR GHQ-12)AND TOPIC: (“common mental disorders” OR CMD OR Anxiety OR anxious OR depression OR dysthymia OR “generalized anxiety disorder” OR “panic disorder” OR phobia OR “social anxiety disorder” OR “obsessive-compulsive disorder” OR “mental disorder” OR “mental health” OR "Psychological stress" OR "Life Stress" OR "Psychologic Stress" OR "Mental suffering" OR Anguish OR "Emotional stress") AND TOPIC:(Survey OR “Cross-sectional studies” OR Prevalence OR frequency OR "Cross-sectional" OR Observational) |
| **LILACS** | (adolescent OR teenager OR child OR young OR teen OR youth OR juvenile OR adolescence OR younger) AND (“general health questionnaire” OR ghq OR ghq-12) AND (“common mental disorders” OR cmd OR anxiety OR anxious OR depression OR dysthymia OR “generalized anxiety disorder” OR “panic disorder” OR phobia OR “social anxiety disorder” OR “obsessive-compulsive disorder” OR “mental disorder” OR “mental health” OR "Psychological stress" OR "Life Stress" OR "Psychologic Stress" OR "Mental suffering" OR anguish OR "Emotional stress") AND (survey OR “cross-sectional studies” OR prevalence OR frequency OR "Cross-sectional" OR observational) AND (collection:("06-national/BR" OR "05-specialized") OR db:("LILACS" OR "MEDLINE")) |
| **GOOGLE SCHOLAR** | With all of the words: adolescent AND GHQ-12  With at least one of the words: “common mental disorders” OR CMD OR Anxiety OR anxious OR depression OR stress  Where my words occurs: anywhere in the article  200 most relevant hits |
| **PROQUEST** | [TI,AB(Adolescent OR Teenager OR Child OR Young OR Teen OR Youth OR Juvenile OR Adolescence OR Younger) AND TI,AB("General Health Questionnaire" OR ghq OR ghq-12) AND TI,AB("common mental disorders" OR cud OR Anxiety OR anxious OR depression OR dysthymia OR "generalized anxiety disorder" OR "panic disorder" OR phobia OR "social anxiety disorder" OR "obsessive-compulsive disorder" OR "mental disorder" OR "mental health" OR "Psychological stress" OR "Life Stress" OR "Psychologic Stress" OR "Mental suffering" OR Anguish OR "Emotional stress") AND TI,AB(Survey OR "Cross-sectional studies" OR Prevalence OR frequency OR "Cross-sectional" OR Observational)](https://search.proquest.com/results.displayspellingsuggestions_0:dospellingsearch?site=pqdtglobal&t:ac=2727255B7108495BPQ/1) |
| **ADOLEC** | (adolescent OR teenager OR child OR young OR teen OR youth OR juvenile OR adolescence OR younger) AND (“general health questionnaire” OR ghq OR ghq-12) AND (“common mental disorders” OR cmd OR anxiety OR anxious OR depression OR dysthymia OR “generalized anxiety disorder” OR “panic disorder” OR phobia OR “social anxiety disorder” OR “obsessive-compulsive disorder” OR “mental disorder” OR “mental health” OR "Psychological stress" OR "Life Stress" OR "Psychologic Stress" OR "Mental suffering" OR anguish OR "Emotional stress") AND (survey OR “cross-sectional studies” OR prevalence OR frequency OR "Cross-sectional" OR observational) AND (instance:adolec) |
| **PsycINFO** | Any Field: Adolescent OR Any Field: Teenager OR Any Field: Child OR Any Field: Young OR Any Field: Teen OR Any Field: Youth OR Any Field: Juvenile OR Any Field: Adolescence OR Any Field: Younger AND Any Field: “General Health Questionnaire” OR GHQ OR GHQ-12 AND Any Field: “common mental disorders” OR CMD OR Anxiety OR anxious OR depression OR dysthymia OR “generalized anxiety disorder” OR “panic disorder” OR phobia OR “social anxiety disorder” OR “obsessive-compulsive disorder” OR “mental disorder” OR “mental health” OR "Psychological stress" OR "Life Stress" OR "Psychologic Stress" OR "Mental suffering" OR Anguish OR "Emotional stress" AND Any Field: Survey OR “Cross-sectional studies” OR Prevalence OR frequency OR "Cross-sectional" OR Observational |
