# Supplementary material for: Common mental disorders prevalence in adolescents: A systematic review and meta-analyses
Source: PLoS One. 2020 Apr 23;15(4):e0232007. doi: 10.1371/journal.pone.0232007 (PMC7179924; doi:10.1371/journal.pone.0232007)
Supplement: S1 Table — (DOC) [file pone.0232007.s003.doc]

# S3 Table. Details of excluded studies.

| **Author, year** | **Tittle** | **Reason for Exclusion** |
| --- | --- | --- |
| Abiodun, 1992 | Mental morbidity in a rural community in Nigeria | Study for instrument validation or exploratory factor analysis |
| Ahnquist, 2010 | What has trust in the health-care system got to do with psychological distress? Analyses from the national Swedish survey of public health | Unable to obtain data exclusively from adolescents |
| Ahnquist, 2011 | Economic hardships in adulthood and mental health in Sweden. the Swedish National Public Health Survey 2009 | Unable to obtain data exclusively from adolescents |
| Ahnquist, 2012 | Social determinants of health e A question of social or economic capital? Interaction effects of socioeconomic factors on health outcomes | Unable to obtain data exclusively from adolescents |
| Allison, 2005 | Relationship of vigorous physical activity to psychologic distress among adolescents | Study presented GHQ data as a continuous variable |
| Amoran, 2012 | Assessment of mental disorders using the patient health questionnaire as a general screening tool in western Nigeria: A community-based study | Study presented GHQ data as a continuous variable |
| Ando, 2017 | Lithium levels in tap water and the mental health problems of adolescents: An individual-level cross-sectional survey | Full-text unavailable |
| Armando, 2010 | Factorial analysis of psychotic like experiences and help seeking behaviour in a community sample of young adults | Study for instrument validation or exploratory factor analysis |
| Åslund, 2014 | The buffering effect of tangible social support on financial stress: influence on psychological well-being and psychosomatic symptoms in a large sample of the adult general population. | Study with a population outside the age group of interest |
| Augustine, 2011 | Perceived stress, life events & coping among higher secondary students of Hyderabad, India: A pilot study | Study presented GHQ data as a continuous variable |
| Bayliss, 2017 | Well-Being During Recession in the UK | Study with specific population |
| Belek, 2000 | Social class, income, education, area of residence and psychological distress: does social class have an independent effect on psychological distress in Antalya, Turkey? | Unable to obtain data exclusively from adolescents |
| Berra, 2006 | Perceived health status and use of healthcare services among children and adolescents | Study with a population outside the age group of interest |
| Biddle, 2004 | What influences help-seeking in mentally distressed young adults? | Unable to obtain data exclusively from adolescents |
| Borrell, 2010 | Perceived discrimination and health by gender, social class, and country of birth in a Southern European country | Unable to obtain data exclusively from adolescents |
| Boyes, 2015 | Adverse Life Experience and Psychological Distress in Adolescence: Moderating and Mediating Effects of Emotion Regulation and Rumination | Study presented GHQ data as a continuous variable |
| Centofanti, 2018 | Establishing norms for mental well-being in young people (7–19 years) using the General Health Questionnaire-12 | Study presented GHQ data as a continuous variable |
| Cheng, 2017 | The effects of family structure and function on mental health during China’s transition: a cross-sectional analysis | Unable to obtain data exclusively from adolescents |
| Chung, 2011 | Assessing insomnia in adolescents: Comparison of Insomnia Severity Index, Athens Insomnia Scale and Sleep Quality Index | Study presented GHQ data as a continuous variable |
| Chung, 2013 | Sleep duration, sleep–wake schedule regularity, and body weight in Hong Kong Chinese adolescents | Study presented GHQ data as a continuous variable |
| Chung, 2014 | Insomnia in adolescents: Prevalence, help-seeking behaviors, and types of interventions | Study presented GHQ data as a continuous variable |
| Crealey, 2018 | Hearing loss, mental well-being and healthcare use: results from the Health Survey for England (HSE) | Study with a population outside the age group of interest |
| Creed, 2002 | Multidimensional Properties of the LOT-R: Effects of Optimism and Pessimism on Career and Well-Being Related Variables in Adolescents | Study presented GHQ data as a continuous variable |
| Cummins, 2005 | Large scale food retailing as an intervention for diet and health: Quasi-experimental evaluation of a natural experiment | Intervention study |
| Dankovicová, 2014 | The impact of life events on mental health of pupils from selected primary schools in the Žilina Region | Study presented GHQ data as a continuous variable |
| Delfabbro, 2011 | Body Image and Psychological Well-Being in Adolescents: The Relationship Between Gender and School Type | Study presented GHQ data as a continuous variable |
| Dorrian, 2018 | Smartphones in the bedroom, sleep, communication, and mental health in australian school students | Study presented GHQ data as a continuous variable |
| Esteban, 2012 | Determinants of poor mental health in people aged 16 to 64 residing in a large city | Unable to obtain data exclusively from adolescents |
| Faria, 2014 | Factors associated with self reported insomnia and sleep loss over worry in young adults | Full-text unavailable |
| Fischer, 2013 | Prevalence and key covariates of non-medical prescription opioid use among the general secondary student and adult populations in Ontario, Canada | Unable to obtain CMD prevalence |
| French, 2004 | Measurement invariance in the General Health Questionnaire-12 in young Australian adolescents. | Study for instrument validation or exploratory factor analysis |
| Fröberg, 2013 | Psychosocial health and gambling problems among men and women aged 16–24 years in the Swedish National Public Health Survey | Unable to obtain data exclusively from adolescents |
| Gaur, 2016 | Mental Health Problems among youth in India and its correlates | Unable to obtain data exclusively from adolescents |
| Gispert, 1998 | Mental health expectancy: An indicator to bridge the gap between clinical and public health perspectives of population mental health | Unable to obtain data exclusively from adolescents |
| Gispert, 2003 | Sociodemographic and health-related correlates of psychiatric distress in a general population | Unable to obtain data exclusively from adolescents |
| Glozah, 2014 | Social support, stress, health, and academic success in Ghanaian adolescents: A path analysis | Study presented GHQ data as a continuous variable |
| Glozah, 2016 | Psychometric Properties of the Perceived Social Support from Family and Friends Scale: Data from an Adolescent Sample in Ghana | Study for instrument validation or exploratory factor analysis |
| Goldman-Mellor, 2010 | Psychological distress and circulating inflammatory markers in healthy young adults | Unable to obtain data exclusively from adolescents |
| Goldney,1991 | Suicidal ideation: its enduring nature and associated morbidity | Unable to obtain CMD prevalence |
| Gooding, 2016 | Assessing social anhedonia in adolescence: The ACIPS-A in a community sample | Study presented GHQ data as a continuous variable |
| Guiney, 2014 | Mental well-being in adolescence and emerging adulthood: a northern ireland perspective | Full-text unavailable |
| Hamilton, 2018 | Psychosocial Health and Lifestyle Behaviors in Young Adults Receiving Renal Replacement Therapy Compared to the General Population: Findings From the SPEAK Study | Study with specific population |
| Hessami, 2013 | Association between cigarette smoking and mental health problems among students | Unable to obtain CMD prevalence |
| Hielscher, 2018 | Do hallucinations predict the transition from suicidal thoughts to attempts? Results from an australian longitudinal cohort study | Unable to obtain CMD prevalence |
| Huuskes, 2016 | Is Belief in God Related to Differences in Adolescents’ Psychological Functioning? | Study with specific population |
| Ismail, 2000 | Do Common Mental Disorders Increase Cigarette Smoking? Results from Five Waves of a Population-based Panel Cohort Study | Unable to obtain CMD prevalence |
| Itani, 2018 | Longitudinal Epidemiologic Study of Poor Mental Health Status in Japanese Adolescents: Incidence of Predic | Unable to obtain CMD prevalence |
| Jaju, 2009 | Prevalence and age of onset distributions of DSM IV mental disorders and their severity among school going Omani adolescents and youths, WMH-CIDI findings | Unable to obtain CMD prevalence |
| Jokela, 2013 | Ageing and the prevalence and treatment of mental health problems | Unable to obtain data exclusively from adolescents |
| Jokela, 2013 | Socioeconomic inequalities in common mental disorders and psychotherapy treatment in the UK between 1991 and 2009 | Unable to obtain data exclusively from adolescents |
| Kaneita, 2007 | Association between mental health status and sleep status among adolescents in Japan: A nationwide cross-sectional survey | Full-text unavailable |
| Kawabe, 2016 | Internet addiction: Prevalence and relation with mental states in adolescents | Study presented GHQ data as a continuous variable |
| Lai, 2009 | Dispositional optimism buffers the impact of daily hassles on mental health in Chinese adolescents | Study presented GHQ data as a continuous variable |
| Lang, 2011 | Income and the midlife peak in common mental disorder prevalence | Unable to obtain data exclusively from adolescents |
| Lewis, 2011 | The Association Between Church Attendance and Psychological Health in Northern Ireland: A National Representative Survey Among Adults Allowing for Sex Differences and Denominational Difference | Unable to obtain data exclusively from adolescents |
| Li, 2010 | Relationships among Mental Health, Self-esteem and Physical Health in Chinese Adolescents | Study presented GHQ data as a continuous variable |
| Li, 2018 | Eveningness chronotype, insomnia symptoms, and emotional and behavioural problems in adolescents | Unable to obtain CMD prevalence |
| Lin, 2011 | The relationship between coping and subclinical psychotic experiences in adolescents from the general population – a longitudinal study | Study presented GHQ data as a continuous variable |
| Lin, 2013 | Psychological Distress, Sources of Stress and Coping Strategy in High School Students | Unable to obtain CMD prevalence |
| López-Castedo, 2005 | Psychometric properties of the Spanish version of the 12-item General Health Questionnaire in adolescents. | Study for instrument validation or exploratory factor analysis |
| Lou Arnal, 1990 | An epidemiological study on mental health in a health area | Full-text unavailable |
| Mahedy, 2013 | Risk factors for psychological distress in Northern Ireland | Unable to obtain data exclusively from adolescents |
| Malmusi, 2011 | Perception or real illness? How chronic conditions contribute to gender inequalities in self-rated health | Unable to obtain data exclusively from adolescents |
| Martins, 2013 | Prevalence of common mental disorders in recently-drafted young Brazilians to mandatory military service and associated factors | Study with specific population |
| Mazur, 2016 | Behavioural factors enhancing mental health − preliminary results of the study on its association with physical activity in 15 to 16 year olds | Study presented GHQ data as a continuous variable |
| McCabe, 1996 | Measuring the mental health status of a population: A comparison of the GHQ-12 and the SF-36 (MHI-5) | Study with specific population |
| Molina, 2014 | Wording effects and the factor structure of the 12-item General Health Questionnaire (GHQ-12) | Study for instrument validation or exploratory factor analysis |
| Moraes, 2018 | The intertwined effect of lack of emotional warmth and child abuse and neglect on common mental disorders in adolescence | Study presented GHQ data as a continuous variable |
| Morales-Carmona, 2008 | Menstrual cycle perception and psychological distress in a Mexican women sample | Unable to obtain data exclusively from adolescents |
| Morelli, 2016 | Sexting, psychological distress and dating violence among adolescents and young adults | Unable to obtain CMD prevalence |
| Ng Fat, 2017 | Evaluating and establishing national norms for mental wellbeing using the short Warwick–Edinburgh Mental Well-being Scale (SWEMWBS): findings from the Health Survey for England | Unable to obtain data exclusively from adolescents |
| Ng, 2010 | Factors related to suicidal ideation among adolescents in Hong Kong | Unable to obtain CMD prevalence |
| Nordmyr, 2014 | Associations between problem gambling, socio-demographics, mental health factors and gambling type: sex differences among Finnish gamblers | Unable to obtain data exclusively from adolescents |
| Okulicz-Kozaryn, 2004 | Diagnosing mental health of adolescents on the basis of their subjective assessments | Full-text unavailable |
| Okwaraji, 2018 | Loneliness, life satisfaction and psychological distress among out-of-school adolescents in a Nigerian urban city | Study with specific population |
| Oliver, 2005 | Help-seeking behaviour in men and women with common mental health problems: cross-sectional study | Unable to obtain data exclusively from adolescents |
| O'Reilly, 2003 | Mental Health in Northern Ireland: Have "The Troubles" Made It Worse? | Unable to obtain data exclusively from adolescents |
| Oskrochi, 2018 | Factors affecting psychological well-being: Evidence from two nationally representative surveys | Unable to obtain data exclusively from adolescents |
| Pantzer, 2006 | Health related quality of life in immigrants and native school aged adolescents in Spain | Unable to obtain CMD prevalence |
| Pevalin, 2000 | Multiple applications of the GHQ-12 in a general population sample: an investigation of long-term retest effects | Unable to obtain data exclusively from adolescents |
| Potard, 2014 | Peer violence, mental health and suicidal ideation in a sample of French adolescent | Study presented GHQ data as a continuous variable |
| Propper, 2005 | Local neighbourhood and mental health: Evidence from the UK | Unable to obtain data exclusively from adolescents |
| Rajmil, 1998 | Prevalence of mental disorders in the general population of Catalonia | Unable to obtain data exclusively from adolescents |
| Réveillère, 2007 | Impact of daily hassles and life events on mental health during preadolescence | Full-text unavailable |
| Ricci-Cabello, 2010 | Mental Disease, Existence of Diagnostic, Use of Psychotropic Medication. Differences by Autonomous Communities under the National Health Survey 2006 | Unable to obtain data exclusively from adolescents |
| Rigby, 2007 | Implications of inadequate parental bonding and peer victimization for adolescent mental health. | Study presented GHQ data as a continuous variable |
| Rocha, 2010 | Prevalence of mental health problems and their association with socioeconomic, work and health variables: Findings from the Spain National Health Survey. | Unable to obtain data exclusively from adolescents |
| Rocha, 2012 | Perception of environmental problems and common mental disorders (CMD) | Unable to obtain data exclusively from adolescents |
| Rocha, 2013 | Inequalities in the Utilization of Psychiatric and Psychological Services in Catalonia: A Multilevel Approach | Unable to obtain data exclusively from adolescents |
| Rocha, 2015 | Inequalities in Mental Health in the Spanish Autonomous Communities: A Multilevel Study | Unable to obtain data exclusively from adolescents |
| Rodríguez-Romo, 2015 | Relationships between physical activity and mental health in the adult population of Madrid | Unable to obtain data exclusively from adolescents |
| Ross, 2017 | Time trends in mental well-being: the polarisation of young people’s psychological distress | Unable to obtain data exclusively from adolescents |
| Ruy-Perés, 2011 | The Relationship Between Reproductive Work and Sociodemographic and Psychosocial Factors in Regard to Psychological Distress in Men and Women in Spain | Unable to obtain data exclusively from adolescents |
| Sabes-Figuera, 2012 | The local burden of emotional disorders. An analysis based on a large health survey in Catalonia (Spain) | Unable to obtain data exclusively from adolescents |
| Sarkova, 2013 | Associations between assertiveness, psychological well-being, and self-esteem in adolescents | Study presented GHQ data as a continuous variable |
| Serrano-Aguilar, 2009 | The relationship among Mental Health Status (GHQ-12), Health Related Quality of Life (EQ-5D) and Health-State Utilities in a general population | Unable to obtain CMD prevalence |
| Seva, 1991 | Health disorders and demand for care in Zaragoza. | Full-text unavailable |
| Shevlin, 2013 | Adolescent loneliness and psychiatric morbidity in Northern Ireland | Study with specific population |
| Shi, 2005 | Epidemiological survey of mental illnesses in the people aged 15 and older in Zhejiang Province , China | Unable to obtain data exclusively from adolescents |
| Shitney, 2018 | Socioeconomic, religious, spiritual and health factors associated with symptoms of common mental disorders: a crosssectional secondary analysis of data from Bhutan’s Gross National Happiness Study, 2015 | Unable to obtain data exclusively from adolescents |
| Stirn, 2006 | Prevalence of tattooing and body piercing in Germany and perception of health, mental disorders, and sensation seeking among tattooed and body-pierced individuals | Study with specific population |
| Sullivan, 2004 | The relationship between adolescent religiosity, spiritual well -being, self esteem and mental illness | Study with specific population |
| Suzuki, 2011 | Clarification of the factor structure of the 12-item General Health Questionnaire among Japanese adolescents and associated sleep status | Study for instrument validation or exploratory factor analysis |
| Tabak, 2004 | Subjective health complaints and psychological distress in adolescents aged 15-19 years in Poland | Full-text unavailable |
| Tabassum, 2016 | Association of volunteering with mental well-being: a lifecourse analysis of a national population-based longitudinal study in the UK | Unable to obtain data exclusively from adolescents |
| Tam, 2011 | The Association between Perceived Social Support, Socio-economic Status and Mental Health in Young Malaysian Adults | Unable to obtain CMD prevalence |
| Taylor, 2007 | The psychological costs of unsustainable housing commitments | Unable to obtain data exclusively from adolescents |
| Tennant, 2007 | The Affectometer 2: a measure of positive mental health in UK populations | Study for instrument validation or exploratory factor analysis |
| Thomas, 2007 | Psychological distress after employment transitions: the role of subjective financial position as a mediator | Unable to obtain data exclusively from adolescents |
| Tochigi, 2016 | Annual longitudinal survey at up to five time points reveals reciprocal effects of bedtime delay and depression/anxiety in adolescents | Study presented GHQ data as a continuous variable |
| Tomcikova, 2009 | Parental Divorce and Adolescent Drunkenness: Role of Socioeconomic Position, Psychological Well-Being and Social Support | Study presented GHQ data as a continuous variable |
| Veselska, 2010 | Socio-economic differences in self-esteem of adolescents influenced by personality, mental health and social support | Study presented GHQ data as a continuous variable |
| Viinamäki, 1995 | The association between economic and social factors and mental health in Finland | Unable to obtain data exclusively from adolescents |
| Wamala, 2009 | How do gender, class and ethnicity interact to determine health status? | Unable to obtain data exclusively from adolescents |
| Wang, 2017 | Epidemiology of severe mental illness in Hunan province in central China during 2014-2015: A multistage cross-sectional study | Unable to obtain data exclusively from adolescents |
| Weich, 1998 | Social roles and gender difference in the prevalence of common mental disorders | Unable to obtain data exclusively from adolescents |
| Weich, 1998 | Poverty, Unemployment, and Common Mental Disorders: Population Based Cohort Study | Unable to obtain data exclusively from adolescents |
| Weich, 1998 | Material standard of living, social class, and the prevalence of the common mental disorders in Great Britain | Unable to obtain data exclusively from adolescents |
| Weich, 2001 | Income inequality and the prevalence of common mental disorders in Britain | Unable to obtain data exclusively from adolescents |
| Weich, 2001 | Social roles and the gender difference in rates of the common mental disorders in Britain: A 7-year, population-based cohort study | Unable to obtain data exclusively from adolescents |
| Weich, 2003 | Contextual risk factors for the common mental disorders in Britain: a multilevel investigation of the effects of place | Unable to obtain data exclusively from adolescents |
| Weich, 2003 | Geographic Variation in the Prevalence of Common Mental Disorders in Britain: A Multilevel Investigation | Unable to obtain data exclusively from adolescents |
| Weich, 2005 | Geographical variation in rates of commonmental disorders in Britain: prospective cohort study | Unable to obtain data exclusively from adolescents |
| Weich, 2006 | Rural/non-rural differences in rates of common mental disorders in Britain: Prospective multilevel cohort study | Unable to obtain data exclusively from adolescents |
| Winzer, 2014 | Positive versus negative mental health in emerging adulthood: a national cross-sectional survey | Study for instrument validation or exploratory factor analysis |
| Zeng, 2014 | Mental health, resilience and core self-evaluation in left-behind rural middle school students | Full-text unavailable |
